# Strategies in Translating the Therapeutic Potentials of Host Defense Peptides

**DOI:** 10.3389/fimmu.2020.00983

**Published:** 2020-05-22

**Authors:** Darren Shu Jeng Ting, Roger W. Beuerman, Harminder S. Dua, Rajamani Lakshminarayanan, Imran Mohammed

**Affiliations:** ^1^Larry A. Donoso Laboratory for Eye Research, Academic Ophthalmology, Division of Clinical Neuroscience, School of Medicine, University of Nottingham, Nottingham, United Kingdom; ^2^Department of Ophthalmology, Queen's Medical Centre, Nottingham, United Kingdom; ^3^Anti-infectives Research Group, Singapore Eye Research Institute, The Academia, Singapore, Singapore

**Keywords:** antibiotic, antimicrobial peptide, antimicrobial resistance, artificial intelligence, host defense peptide, nanoparticle, peptide

## Abstract

The golden era of antibiotics, heralded by the discovery of penicillin, has long been challenged by the emergence of antimicrobial resistance (AMR). Host defense peptides (HDPs), previously known as antimicrobial peptides, are emerging as a group of promising antimicrobial candidates for combatting AMR due to their rapid and unique antimicrobial action. Decades of research have advanced our understanding of the relationship between the physicochemical properties of HDPs and their underlying antimicrobial and non-antimicrobial functions, including immunomodulatory, anti-biofilm, and wound healing properties. However, the mission of translating novel HDP-derived molecules from bench to bedside has yet to be fully accomplished, primarily attributed to their intricate structure-activity relationship, toxicity, instability in host and microbial environment, lack of correlation between *in vitro* and *in vivo* efficacies, and dwindling interest from large pharmaceutical companies. Based on our previous experience and the expanding knowledge gleaned from the literature, this review aims to summarize the novel strategies that have been employed to enhance the antimicrobial efficacy, proteolytic stability, and cell selectivity, which are all crucial factors for bench-to-bedside translation of HDP-based treatment. Strategies such as residues substitution with natural and/or unnatural amino acids, hybridization, L-to-D heterochiral isomerization, C- and N-terminal modification, cyclization, incorporation with nanoparticles, and “smart design” using artificial intelligence technology, will be discussed. We also provide an overview of HDP-based treatment that are currently in the development pipeline.

## Introduction

Antimicrobial resistance (AMR) currently represents a major global health threat of the twenty-first century and is estimated to cost 10 million deaths per year by 2050 (1, 2). Tackling AMR requires a multi-faceted and synergistic approach, including understanding of the main mechanisms and drivers of AMR at the microorganism, individual and population levels, antimicrobial stewardship within the healthcare and agriculture sectors, in tandem with discovery and development of new classes of antimicrobial therapy (3, 4).

Host defense peptides (HDPs), also known as antimicrobial peptides, are a group of evolutionary conserved molecules that play critical roles in the innate immune system (5–7). These molecules are usually small in size (around 12–50 amino acid residues), cationic (with a net charge of +2 to +13), and amphiphilic geometry (5, 6). So far, more than 3,000 naturally occurring and synthetic HDPs have been discovered across six life kingdoms (8, 9), underlining their essential roles during the adaptation and evolutionary process. HDPs have been shown to exhibit broad-spectrum antimicrobial activities against a plethora of microorganisms, including drug-resistant bacteria, fungi, parasites, and viruses (5, 10). In contrast to conventional antibiotics (which usually target a particular extracellular or intracellular binding site), cationic HDPs bind to the anionic surfaces of bacteria causing lytic cell death through varied mechanisms. Additionally, HDPs could also kill bacteria via inhibition of macromolecules that are involved in the biosynthesis of the surface membrane, and components of metabolic organelles (11, 12). Such unique mechanism of action accounts for the rapid and broad-spectrum antimicrobial action and the low risk of developing AMR as modification of the entire microorganisms' anionic membrane incurs a substantially higher fitness cost when compared to alteration of a particular binding site targeted by the conventional antibiotics (e.g., alteration in the 30S subunit inhibits the action of aminoglycosides) (13).

In addition to the antimicrobial activity, there has been emerging evidence highlighting the non-antimicrobial roles of HDPs, including antibiofilm (14), immunomodulatory (15), wound healing (16), anti-viral, and anti-cancer properties (17), rendering them an attractive and novel class of clinical therapeutics (18). For instance, corneal infection or infectious keratitis is a sight-threatening ocular disease that can be caused by a wide range of microorganisms, including bacteria, fungi, parasites, and viruses (19, 20). In addition, corneal infection may be caused by polymicrobial infection which can present significant therapeutic challenges to the clinicians (21). The dual broad-spectrum antimicrobial and wound healing properties of HDPs would not only provide a comprehensive antimicrobial coverage for the infection but also expedite the healing process of corneal ulceration, limiting the damage to the cornea and ultimately preserving the vision. The complete repertoire of antimicrobial and non-antimicrobial functions of HDPs has been recently summarized in a seminal review by Mookherjee et al. (22).

Despite decades of research efforts have been invested in the field of HDP, the mission of translating novel HDP-derived molecules from bench to beside has yet to be fully accomplished. The sluggishness in the HDP-derived drug development pipeline is primarily attributed to their complex structure-activity relationship (SAR), undesired toxicity to host tissues, instability in host and microbial environment, lack of correlation between *in vitro* and *in vivo* efficacies, and dwindling interest from large pharmaceutical companies (23–26). A list of synthetic derivatives of HDPs that are currently under clinical trials are summarized in Table 1 for readers' convenience. Detailed overview on the mechanisms of actions and clinical applications of HDPs have also been provided elsewhere in published review articles (27–29). Based on the literature and our experience, this review article aims to highlight the strategies that have been attempted to enhance the antimicrobial efficacy, proteolytic stability, and cell selectivity (for microbial cells instead of host cells; i.e., improved safety) of HDPs, which are all important factors to be considered during the translation of these molecules.

**Table 1:** Derivatives of host defense peptides in clinical trials.

**Drug/peptide name**	**Peptide derivative**	**Progress**	**Application**	**References/Trial registry (Status; Last update posted)**
Omiganan (CLS001/MBI226)	Indolicidin	Phase II/III	Facial seborrheic dermatitis; Genital warts; Rosacae; Vulvar neoplasia; Atopic dermatitis; Acne vulgaris; Skin antisepsis; Prevention of catheter infections	NCT03688971 (recruiting; May 2019) NCT02576847 (completed; Jul 2018) NCT02547441 (completed; Jul 2018) NCT02576860 (completed; Mar 2018) NCT03091426 (completed; Dec 2017) NCT02596074 (completed; Aug 2017) NCT03071679 (completed; May 2017) NCT02571998 (completed; Mar 2017) NCT02849262 (completed; Mar 2017) NCT02456480 (completed; Jul 2016) NCT02066545 (completed; Sept 2015) NCT01784133 (completed; May 2015) NCT02028286 (completed; Apr 2014) NCT00608959 (completed; Jan 2010) NCT00231153 (completed; Aug 2009) NCT00027248 (completed; Sept 2005)
Pexiganan (MSI-78)	Magainin	Phase III	Diabetic foot ulcer (topical)	NCT01594762 (completed; Jun 2017) NCT01590758 (completed; Jun 2017) NCT00563433 (completed; Nov 2007) NCT00563394 (completed; Nov 2007)
Iseganan (IB-367)	Protegrin	Phase II/III	Prevention of radiation-induced oral mucositis; Ventilator-associated Pneumonia	NCT00022373 (unknown; Oct 2014) NCT00118781 (terminated; Jul 2005) (failed in Phase III; https://www.thepharmaletter.com/article/intrabiotics-lead-drug-hits-snag-in-phase-iii)
C16G2	Novispirin G10	Phase II	Dental cavities	NCT02594254 (completed; Aug 2019) NCT02509845 (completed; Aug 2019) NCT03196219 (completed; Aug 2019) NCT02254993 (completed; Aug 2019) NCT03052842 (completed; Aug 2019) NCT03004365 (completed; Aug 2019) NCT02044081 (completed; Aug 2019)
Brilacidin (PMX-30063)	Defensin mimetic	Phase I/II	Prevention of radiation-induced oral mucositis, treatment of gram-positive bacterial skin infections	NCT04240223 (completed; Feb 2020) NCT02324335 (completed; Jan 2019) NCT02052388 (completed; Sep 2018) NCT01211470 (completed; May 2012)
Novexatin (NP213)	Polyarginine cyclic HDP	Phase II	Onychomycosis (topical)	www.novabiotics.co.uk (no registered trial)—PMID 32232410
PAC-113	Histatin-3	Phase IIb	Oral candidiasis (mouth rinse)	NCT00659971 (completed; Jun 2008)
hLF_1−11_	Human lactoferrin	Phase II	Bacteraemia; Candidemia; anti-infectives for haematopoietic stem cell transplant recipients	NCT00430469 (withdrawn; Jun 2015) NCT00509834 (withdrawn; Jun 2015) NCT00509847 (withdrawn; May 2014) NCT00509938 (completed; Oct 2008)
OP-145 (AMP60.4Ac)	Human LL-37	Phase II	Chronic suppurative otitis media	ISRCTN12149720 (completed; Feb 2019) ISRCTN84220089 (completed; Jan 2008)
Glutoxim (NOV-002)	Synthetic hexapeptide	Phase II/III	Tuberculosis; myelodysplastic syndromes; ovarian cancer; non-small cell lung cancer; breast cancer;	NCT00960726 (withdrawn; Jul 2012) NCT00499122 (completed; Jan 2018) NCT00347412 (completed; Nov 2011) NCT00345540 (completed; Mar 2015)
Lytixar (LTX-109)	Synthetic peptidomimetic	Phase I/IIa	Impetigo; drug-resistant gram-positive nasal and skin infections (topical); atopic dermatitis	NCT01158235 (completed; Jun 2011) NCT01223222 (completed; Feb 2011) NCT01803035 (completed; Apr 2014)
Opebacan (rBPI-21)	Bactericidal/permeability increasing protein (BPI)	Phase I/II	Prevention of endotoxemia following myeloablative allogeneic stem cell transplantation; anti-sepsis in patients with burn injury	NCT00454155 (terminated; Jul 2012) NCT00462904 (terminated; Nov 2019)

## Strategies for Enhancing Antimicrobial Efficacy and Safety of HDPs

Structurally, HDPs are mainly characterized by the presence of key charged residues (e.g., Arg and Lys), with a high proportion of hydrophobic residues (constituting 50% or more) and amphipathicity (7, 30). Optimization of HDP sequences to improve antimicrobial efficacy has been previously detailed (30, 31). Here, we aim to provide key strategies to improve the selectivity of HDPs toward microbial membranes through reported examples of model HDPs that were enhanced through rational design approaches (Table 1).

### Residue Substitution With Natural Amino Acids (AAs)

HDPs should fulfill key functional requirements to qualify for clinical use, including low toxicity, high antimicrobial activity, and good *in vivo* stability (24). These requirements are closely linked to their biochemical selectivity toward anionic and zwitterionic surfaces (24). The antimicrobial activity is attributed to a fine balance of hydrophobicity, cationic residues, amphipathicity, and structural conformation (e.g., α-helical, β-sheet, and cyclized) (32). On the other hand, the hydrophobic interaction between specific residues of HDPs (e.g., Leu, Ile, Val, Phe, Tyr, Trp) and zwitterionic phospholipids on host cell surfaces is responsible for its toxicity. For example, peptide derivatives of mastoparan (a key constituent of wasp venom) that were designed based on fixed five rules utilizing the quantitative structure-activity relationship (QSAR) approach showed potent antimicrobial efficacy against *Bacillus subtilis* (33). It was shown that the potency of these derivatives was mainly dependent on the presence of Trp, Lys, and His (33). Lata et al. (34) analyzed 486 HDPs from the antimicrobial peptide database (APD) for AA frequency in these sequences using the bioinformatic tools. Residues such as Gly, Arg, Lys, and Leu were shown to be commonly found in HDPs, whilst AAs such as Ser, Pro, Glu, and Asp were least common at both N- and C-terminus. A recent study from Hilpert group has demonstrated through *in silico* designed library of 3,000 *de novo* short peptides (9-mer in length) that the specific design characteristics of HDPs did not apply to short peptides (35). The peptide sequences that were grouped as “super active” based on their activity toward *Pseudomonas aeruginosa* were mainly composed of Lys, Arg, Trp, and Val/Ile/Leu (35). However, the activity of these super peptides toward host cells and *in vivo* stability was not yet reported.

Melittin, a key constituent of honey-bee venom, is a potent HDP with strong antimicrobial activity (36). However, its clinical use is largely limited by the high hemolytic activity (36). Blondelle et al. (37) studied the function of Trp in melittin activity through serial Trp substitution starting from N- to C-terminus. Substitution of Leu → Trp at 9th position was shown to decrease the hemolytic activity whereas substitution of Pro → Trp at 14th position improved the alpha-helical conformation and reduced the hemolytic effects compared to parent melittin peptide.

HDPs with Pro residues are widely known to display a disrupted helix conformation, which eventually affects their surface retention time and penetration into microbe cytoplasm (38, 39). A recent study using peptide analog (Anal 3, 19-AA long) from N-terminus of *Helicobacter pylori* ribosomal protein L1 has demonstrated that an insertion of Pro-hinge into Anal 3 (via Glu → Pro substitution at 9th position) significantly improves the peptide selectivity toward microbes with no effect on host cells (40, 41). This was attributed to the helix-hinge-helix conformation of Anal 3-Pro analog at the surface of bacteria allowing peptide penetration and DNA binding in the cytoplasm. This study suggested that rational insertion of Pro residue through SAR analysis could improve the biological membrane selectivity of microbicidal peptides. Proline-rich designed HDPs such as ARV-1502 (42), oncocin (43), and Bac-5 (44, 45) have shown significant efficacy against Gram-negative pathogens but not host cells membranes. Unlike cationic HDPs, proline-rich peptides kill bacteria through inhibition of protein synthesis (12, 45–47). Histidine-rich (48, 49), alanine-rich (50), and tryptophan-rich (51) short HDPs have also been developed. These were shown to be highly effective at acidic pH against a range of Gram-negative and Gram-positive bacteria (52).

Magainin-2 is 23 residues long antimicrobial peptide (AMP) isolated from frog skin, *Xenopus laevis* (53). Due to its non-hemolytic and broad-spectrum antimicrobial properties, magainin-2 was widely studied as a model peptide to understand the SAR of naturally occurring AMPs (54–58). Chen et al. (59) have demonstrated that the alpha-helical conformation of magainin-2 could be stabilized through Gly → Ala substitution (at both 13th and 18th position) and C-terminal amidation. This was shown to increase the antibacterial activity by 2-fold against a range of bacteria without modulating its safety against erythrocytes (59). Numerous groups have made attempts to improve the activity of magainin-2 against Gram-negative bacteria through residue substitution. It was demonstrated that the substitution of Phe → Trp in magainin-2 (F12W mutant) increased its activity against Gram-negative bacteria; however, this increased its selectivity toward erythrocytes, causing significant hemolysis (60). This could be attributed to the bulkiness of Trp compared to Phe and the presence of NH-group in Trp that is capable of forming hydrogen bonds with zwitterionic phospholipids (39, 61). Further modification through reduction of net charge of F12W mutant (Lys → Glu substitution at 10th position) was shown to reduce the hemolytic effect. However, this made the mutant magainin-2 less effective against Gram-negative bacteria (60). Extensive SAR studies from Zasloff's laboratory led to the development of MSI-78 (also known as pexiganan), a derivative of magainin-2, which showed improved safety/efficacy profile compared to parent magainin-2 sequence (62, 63). However, it failed in the phase III clinical trial for the treatment of infective diabetic foot ulcers. Further details on pexiganan clinical journey can be reviewed elsewhere (64–66).

Typically, natural HDPs display a net positive charge between +2 and +13. It has been widely demonstrated that modification of total net charge of synthetic HDPs through cationic residue substitution enhances electrostatic interaction between HDPs and lipopolysaccharide (LPS) (30, 60). However, this approach has shown to increase the toxicity of certain HDPs. For example, magainin-2 analogs with positive charge above +5 were shown to display hemolytic effects (rank order +6 > +5 > +4 > +3) (67). To overcome the inherent issues associated with peptide optimization, Mishra and Wang (68) adopted an *ab initio* design approach which involved utilization of novel database-filtering technology (DFT). This led to the development of a 13-AA long, leucine-rich, anti-MRSA peptide template—termed “DFTamP1” (68). A subsequent study demonstrated that DFT503, an optimized variant of DFTamP1, was shown to be safe and effective in *in vivo* killing of MRSA in a neutropenic mouse model. This anti-MRSA activity was attributed to its eight Leu residues and a single Lys at position 11 (net charge +1) (69). These studies suggested that lower cationic charge and high hydrophobicity is preferred for anti-MRSA synthetic peptides. This strategy could form the basis for the development of species-specific peptide-based therapy against multidrug resistant (MDR) pathogens.

LL-37 is a lone member of the cathelicidin family of HDPs reported in humans (70–72). It was widely studied due to its multi-functional abilities, including microbicidal (73), anti-cancer, immunomodulatory (74), chemotactic (75), and wound-healing properties (76). Numerous groups have exploited the structure of LL-37 to design a range of synthetic antimicrobial analogs through residue substitution (77, 78). FK-13 (residues 17-29 of LL-37) was identified as a core antimicrobial and anti-cancer domain using nuclear magnetic resonance (NMR) technique (79). Subsequently, the deletion of Phe at 17th position led to the development of KR-12, which showed potent antimicrobial efficacy equivalent to LL-37 and FK-13 against *Escherichia coli*, but devoid of toxic activity against host cells (80). KR-12 and KE-18 analogs were recently shown to possess anti-*Candida* and anti-*Staphylococcal* properties (81). Specifically, KE-18 showed anti-biofilm activity even at sub-killing concentration against yeast and bacteria (81). Further variants of KR-12 were also reported and the less cationic analogs, a5 and a6, were shown to possess potent immunomodulatory, antibiofilm, antimicrobial, and osteogenic properties (82–85). Variants of LL-23, corresponding to 23 N-terminal residues of LL-37, were generated through substitution of Ser → Ala and Ser → Val at the 9th position. LL-23V9 peptide was shown to display increased antimicrobial and immunosuppressive activities compared to LL-23 and parent LL-37 (86). Wang et al. (77), Mishra and Wang (87) have recently demonstrated that titanium surface immobilized FK-16 (a short variant of LL-37) is highly antimicrobial against ESKAPE pathogens. Our group has recently demonstrated that FK-16 could be used for repurposing conventional antibiotics such as vancomycin as a strategy to counter antimicrobial resistance (88). Further improvement of FK-16 by Narayana et al. (89) have also led to the development of GF-17, 17BIPHE2, and other related variants of superior efficacy and safety compared to LL-37. Nell et al. (90) designed a range of short peptides through residue substitution based on the LL-37 sequence for neutralization of lipopolysaccharide (LPS) and lipoteichoic acid (LTA). P60.4, a 24-AA derivative, was shown to possess similar LPS/LTA neutralization ability and antimicrobial effects compared to LL-37, but with negligible *in vivo* toxicity toward audible canal, skin, and eyes. This peptide was subsequently termed as OP-145 and was proven to be safe and efficacious in the treatment of chronic otitis media in phase I/II clinical trials (91). However, the activity of OP-145 was recently shown to be reduced in human plasma (92). Subsequent modification led to the development of synthetic antimicrobial and antibiofilm peptides (SAAPs) such as SAAP-145, −148, and −276, which showed potent anti-biofilm activity against a range of MDR pathogens (92).

### Residue Substitution With Unnatural AAs

HDPs are essentially a group of small bioactive molecules made of different combinations of 20 naturally occurring AAs. The nearly infinite chemical space (20^n^) and varying physicochemical properties account for the vast structural and functional diversities of naturally occurring HDPs (8, 9). However, susceptibility to host cell interaction (e.g., human erythrocytes, albumin, etc.) (93, 94) and proteolytic degradation from the host and bacterial proteases (e.g., human proteases in serum, staphylococcus aureolysin, pseudomonas elastase, etc.) (95–98) remains one of the key impediments in translating HDP-based treatment to clinical therapeutics. For instance, the anti-staphylococcal activity of cathelicidin (LL-37)—one of the most widely studied human HDPs—was shown to be inhibited by the proteases produced by *Staphylococcus aureus*, namely the aureolysin (a metalloproteinase) and V8 protease (glutamylendopeptidase), via cleavage and hydrolysis of the intramolecular peptide bonds (96).

To overcome this barrier, incorporation of unnatural or non-proteinogenic AAs has been employed to increase the proteolytic stability and/or antimicrobial efficacy of HDPs. It is known that the antimicrobial efficacy of HDPs is greatly influenced by the cationicity (99). To preserve the cationicity and thence efficacy, researchers have attempted to optimize the HDPs by replacing the cationic residues (e.g., lysine) with its analogs such as ornithine, 2,4-diamino-butyric acid (DAB), and 2,3-diamino-propionic acid (DAP), which have three, two, and one methylene (CH_2_) groups in the side chain, respectively (100, 101). Using Trp-rich peptides as the design template, Arias et al. (101) reported a 4-fold length-dependent increase in the antibacterial activity against *E. coli* when the side chain of lysine was shortened from 4-carbon (lysine) to 1-carbon (DAP). Such effect was likely attributed to an increase in membrane permeabilization based on calcein leakage study. In addition, a substantial improvement in the stability against trypsin was observed when the side chain of arginine or lysine was shortened (101). Oliva et al. (102) investigated the potential role of integrating unnatural AAs within the 9-residue synthetic HDPs and discovered that unnatural AAs such as 2-naphthyl-L-alanine (an aromatic residue) and S-tert-butylthio-L-cysteine residues enhanced the antimicrobial efficacy and proteolytic stability in 10% serum for 1 and 16 h (to a lesser extent). In addition, incorporation of unnatural AAs dipeptides (tetrahydroisoquionolinecarboxylic acid-octahydroindolecarboxylic acid; or Tic-Oic) within magainin analogs has been shown to induce an amphipathic and loose alpha-helical structure with enhanced antimicrobial potency and selectivity against Gram-positive, Gram-negative and mycobacterium (103).

Unnatural AAs have been successfully utilized for improving the efficacy and stability of various peptidomimetics (104). For example, Saralasin, a partial angiotensin II receptor agonist, was developed by incorporation of sarcosine (an unnatural AA) at a key position in angiotensin II molecule (105). This provided resistance against aminopeptidases and improved bioactivity. Carbetocin, a cyclic 8-AA derivative of oxytocin is currently used for the treatment of post-partum hemorrhage, was developed through incorporation of unnatural AAs such as methyltyrosine which improved its metabolic stability and overall therapeutic benefits (106).

### Hybridization

It is widely known that a cocktail of HDPs are produced at the tissue sites in response to infection (107, 108). This natural synergism between HDPs was shown to be beneficial to the host, providing the first line of defense against pathogens. This was very well-exploited through *in vitro* and *in vivo* studies, which proved that the combination of two HDPs produces strong activity against bacteria (107, 108). However, this was not deemed as a cost-effective approach and the issue of host toxicity remains unresolved. Hybridization strategy was shown to circumvent these known issues, which involves the combination of key residues from two to three HDPs of different mechanisms of actions into a single sequence (109–111). In 1989, Boman et al. (112) elegantly showed that a hybrid of cecropin-A (1-13) and melittin (1-13) was highly bactericidal and less toxic toward host cells compared to parent cecropin-A and melittin. Subsequent modifications led to the development of numerous short hybrids of cecropin-A and melittin (15–18 residues in length), which showed similar activity as the first-generation hybrids (113, 114). Chimeras of cecropin-A (CA) and magainin-2 (MA) were also developed that exhibited potent antibacterial and antitumor activities. Insertion of hydrophobic residues through residue substitution in the hinge region (at 16th position) of CA(1-8)-MA(1-12) hybrid was shown to improve its antibacterial and antitumor activity with no hemolytic effects (115). A recent study has demonstrated that substitution of key residues in CA(1-8)-MA(1-12), specifically Phe5Lys, Lys7His, Phe13His, Leu14Phe, and His17Leu, could stabilize the alpha-helical conformation, resulting in improved LPS binding affinity, increased bactericidal activity against clinical Gram-negative isolates, and low cytotoxicity (116). Hybrids of human-derived and animal-derived HDPs were also developed to comprise the membrane-lytic and immunomodulatory properties of cationic HDPs. For example, hybrids of cecropin-A (1-8)-LL37(17-20) (117), melittin(1-13)-LL37(17-30) (118), and BMAP27(9-20)-LL37(17-29) (119) were shown to be highly bactericidal and improved the efficacy of conventional antibiotics against a variety of bacteria. Another study involving “triple hybrid” of cecropin-A, melittin, and LL-37 showed that this approach could significantly enhance the bactericidal against a range of Gram-negative and Gram-positive organisms (120). Similarly, Dutta et al. (121) reported good *in vitro* and *in vivo* efficacies of an antimicrobial contact lens coated with melimine (derived from melittin and protamine) for treating infectious keratitis in a rabbit model. These studies have clearly indicated that an optimized rational design approach could enable development of chimeras with improved biological selectivity.

In addition to overcoming the issue of host toxicity (as described above), the hybrid strategy has also led to the development of numerous species-specific and targeted bactericidal peptides to prevent damage to useful microbiome (122–124). Kim et al. (122) have developed a targeted chimeric peptide for the treatment of *P. aeruginosa* infection. Through phage-display library screening, they identified an outer-membrane porin F (OprF) binding peptide motif, termed PA2. Hybridization of this tag sequence to a membrane-lytic short peptide, GNU-7, was shown to improve the antimicrobial efficacy of parent GNU-7 by 16-fold toward *P. aeruginosa* both in *in vitro* and *in vivo* model systems. LPS-targeting GNU-7 variants were also developed through hybridization with lactoferrin (28-34), BPI (84-99), and *de novo* sequence (125). Chimeric bactericidal peptides targeted to *E. faecalis* (123) and *S. mutans* (124) were also developed based on the species-specific pheromones. This approach was proven to be highly targeted and would prevent the damage to commensal microbes.

### L-to-D Heterochiral Isomerization

Depending on the geometric arrangement, all naturally occurring AAs (except for glycine) can exist as stereoisomers, either in L- or D-form, albeit only the L-configuration can be utilized by cells (126). That said, there is emerging evidence showing that most organisms are able to produce D-AAs, primarily through spontaneous racemization of L-AAs or post-translational enzymatic modification (127). In addition, D-AAs such as D-alanine and D-glutamic acid are found in peptidoglycan, which is a key component of the cell wall of Gram-positive bacteria. These D-AAs have been shown to increase resistance to host proteases that usually cleave the peptide bonds between L-AAs, thereby maintaining their virulence (126).

Capitalizing on the evolutionarily advantageous strategy equipped by microbes, L-to-D isomerization has been utilized to enhance the proteolytic stability of HDPs against a range of host and microbes' proteases (128–133). L-to-D isomerization can be utilized to either modify specifically one or several L-AAs (131, 134), or the entire sequence of a L-form HDP (130–132). Carmona et al. (130) demonstrated that L-to-D isomerization of Panidin-2 (D-Pin2) improved the cell selectivity (i.e., reduced hemolysis) and proteolytic stability in human serum, elastase, and trypsin, while maintaining the antimicrobial activity against a range of Gram-positive and Gram-negative bacteria. In a similar vein, Jia et al. (131) reported an improved stability of D-AA derivative of polybia-CP (which was originally derived from the venom of social wasp *Polybia paulista*), in chymotrypsin and trypsin for 1 and 6 h and reduced hemolytic activity (D-lysine derivative). In addition to the beneficial effect of proteolytic stability and/or cell selectivity, the D-form AAs may enhance the antimicrobial efficacy of HDPs. For example, the D-form KLKLLLLLKLK-NH2 (derived from sapesin B) was shown to exhibit increased antimicrobial efficacy against *S. aureus* (due to increased interaction with the peptidoglycan), *E. coli* and *Candida albicans* when compared to the L-form (132). However, the enhanced antimicrobial efficacy was not observed in other tested D-forms of HDPs such as mastoparan M and temporin A, suggesting that the D-isomerization effect is sequence-dependent (132).

L-to-D isomerization has also been shown to confer unique changes to the peptide-folding and secondary structure of HDPs (128, 131, 133). Based on circular dichroism analysis, the D-form derivatives of naturally occurring alpha-helical HDPs typically exhibit a left-hand alpha-helical spectrum (instead of a right-hand spectrum) whereas partial D-isomerization of HDPs may result in some degree of loss of alpha-helicity, depending on the position and number of D-AAs being introduced (128, 131, 133). Such changes in the secondary structure are likely accountable for the reduced host toxicity and improved cell selectivity in some HDPs (131).

This strategy was found to be successful in the development of daptomycin, an antibacterial cyclic lipopeptide, which was approved by the US Food and Drug Administration (FDA) in 2003 for the treatment of skin and systemic Gram-positive infections (104, 135). Structurally, daptomycin is comprised of 13 residues including D-alanine and D-serine (134). In addition, it also contains non-canonical amino acids such as ornithine, L-kynurenine, and L-3-methylglutamic acid (134).

### C- and N-Terminal Modification

A range of N- and C-terminal modification strategies have been proposed to enhance the antimicrobial efficacy and/or cell selectivity of natural and synthetic HDPs (102, 136, 137). Amongst all, N-terminal acetylation (CH_3_CO-) and C-terminal amidation (-NH_2_) are the two most commonly attempted strategies (102). N-acetylation is a common protein modification observed in eukaryotic and prokaryotic cells (138). By neutralizing the positive charge (NH${}_{3}^{+}$) at the N-terminal, N-acetylation can result in a range of irreversible changes to the protein properties, including the folding, stability, and protein-protein interactions (138). Saikia et al. (139) examined the antimicrobial efficacy and salt sensitivity of *E. coli*-derived MreB (a bacterial cytoskeleton protein found in non-spherical cells) and its N-acetylated analogs and found that N-acetylated W-MreB_1−9_ demonstrated a higher antimicrobial efficacy (in salt) compared to W-MreB_1−9_. However, N-acetylation may result in a decrease in antimicrobial efficacy of certain synthetic HDPs due to a reduction in the overall cationicity (139, 140), suggesting that the benefit of this modification strategy is only selective for certain HDPs.

On the other hand, C-amidation is a common post-translational modification that is widely observed in nature, including the natural synthesis of HDPs (137). C-amidation has been shown to improve the antimicrobial efficacy of certain HDPs, including aurein (141), melittin (142), modelin-5 (143), anoplin (144), and esculentin-1 (145), amongst others. The enhanced antimicrobial efficacy of these HDPs is likely ascribed to the increased alpha-helix stability at the peptide-membrane interfaces, enabling a greater membrane disruption and pore formation (141–144). In addition, Oliva et al. (102) demonstrated that simultaneous N-acetylation and C-amidation enhanced the proteolytic stability of HDP derived from human apolipoprotein B by more than 4-fold when exposed to fetal bovine serum 10% for 1 h. Similarly, the proteolytic stability of tachyplesin I (a beta-hairpin HDP from the horseshoe crab, *Tachypleus tridentatus*) in fresh human serum was significantly enhanced using the similar N-acetylation and C-amidation strategy (146).

Other N- or C-terminal modification strategies have also been described in the literature, including N-methylation of certain cyclic HDPs to enhance the antimicrobial efficacy (136), introduction of 6-aminocaproic acid at the N- and C-terminals to protect HDPs from the action of exopeptidases (102, 147), and pegylation of the C-terminus of M33, a branched peptide, to increase the resistance against *P. aeruginosa* elastase (148).

### Cyclization

Cyclization is a common phenomenon observed in natural HDPs that can exist in three main forms: (a) sidechain to sidechain; (b) backbone to backbone; and (c) sidechain to backbone (137). It has been shown to demonstrate several favorable biological properties, including enhanced antimicrobial efficacy, stability against proteases (due to conformational rigidity), enhanced cell selectivity, and reduced host toxicity (137, 149), rendering it an attractive strategy for translating HDPs from bench to bedside. Some of the notable examples of cyclic glyco- or lipopeptides that are already in clinical use include vancomycin, daptomycin, and colistin/polymyxin, which are commonly used as last resorts for combatting MDR bacteria, albeit their widespread use are hindered by the inherent toxicity and emergence of AMR (149–151).

In view of the structural stability, Dathe et al. (152) were able to create a series of short cyclic hexapeptides (based on AcRRWWRF-NH_2_) with enhanced antimicrobial efficacy (up to >16-fold increase) against *Bacillus subtilis* and *E. coli* compared to the linear form, though the hemolytic activity was increased by 3-fold (152). It was also found that the antimicrobial activities of those small Arg/Trp-rich cyclic peptides were influenced by the self-assembling behavior of peptides at the bacterial membrane instead of their hydrophobic surface area, amphiphilicity, and ring size (153). In addition, a number of small cyclic D,L-alpha-peptides (with six or eight alternating D- and L-form residues) have also demonstrated strong antimicrobial efficacy against Gram-positive and/or Gram-negative bacteria via self-assembly on the bacterial membranes as organic nanotubules, which could increase membrane permeability and disrupt transmembrane ion potentials with resultant cell lysis (154, 155). Furthermore, molecular dynamic simulations and biophysical assays have provided further supportive evidence that cyclic peptides are able to bind to negatively charged membrane more strongly than the linear peptides and adopt a beta-sheet structure at the membrane surface (156).

Another form of cyclization that is found abundantly in natural HDPs, mainly in defensins, is the disulfide intramolecular cross-link between cysteine residues, which has been shown to enhance proteolytic stability (157–160). Inspired by the nature, Scudiero et al. (160) engineered a 17-residue cyclic synthetic hybrid HDP, based on the internal hydrophobic domain of human-beta defensin (HBD)-1 and positively charged C-terminal of HBD-3 (RRKK residues), and demonstrated good antimicrobial efficacy against Gram-positive and Gram-negative bacteria and herpes simplex virus, with low toxicity and good proteolytic stability. Similarly, Mwangi et al. (161) successfully developed a cyclic HDP-based molecule called ZY4 by introducing a disulfide bond to a derivative of cathelicidin-BF, which is an antimicrobial peptide derived from the snake venom of *Bungarus fasciatus*. This molecule was shown to exhibit significant *in vivo* antimicrobial efficacy against MDR *P. aeruginosa* and *A. baumannii* with high stability in mice lung infection and septicemia models (161).

### Incorporation With Nanoparticles (NPs)

Nanotechnology is a rapidly growing field in biotechnology that involves characterization, manipulation and synthesis of materials that are in nanoscales (or one billionth of meter; 10^−9^ m) (162). NPs, with sizes ranging from 1 to 100 nm, can exist in many forms, including lipid-based, metal-based, carbon-based, ceramics, semiconductor, and polymeric NPs (163). It has increasingly been applied in the field of antimicrobials, either employed as antimicrobial agents or nano-carriers for drug/peptide delivery in view of their enhanced protection against extracellular degradation, improved bioavailability, and cell selectivity (164–167). Recently, Biswaro et al. (166) have provided an excellent review on the role of nanotechnology in delivering HDPs. To avoid any significant overlap, this section aims to only recapitulate the fundamental principles of NPs and provide some notable examples regarding the potential values of incorporating NPs with HDPs.

In principle, there are two types of nano-delivery systems: (a) passive delivery where the intended drugs/peptides are encapsulated within the nanocarriers through hydrophobic interaction without any surface modification; and (b) active delivery where the drugs/peptides are directly conjugated with the nanocarriers with surface modification with ligands or other moieties to facilitate delivery to the targeted site (168). Among all, LL-37 and its mimics are some of the most commonly explored HDPs that have been incorporated with different types of NPs, including polymeric NPs [e.g., poly lactic-co-glycolic acid (PLGA)] (169), gold NP (170, 171), and magnetic NPs (172).

PLGA is a FDA-approved biodegradable and biocompatible polymer that has demonstrated promising potential as a drug delivery carrier (173). Chereddy et al. (169) described using PLGA as a nanoparticle carrier for delivering a sustained release of LL-37 treatment. Compared to PLGA or LL-37 alone, PLGA-LL37 nanoparticles were reported to expedite the wound healing process with significantly higher collagen deposition, re-epithelialization and neovascularization (169). It also demonstrated better antimicrobial activity against *E. coli* compared to PGLA alone, though the efficacy was lower than LL-37 alone (169). Cruz et al. (174) similarly reported that the encapsulated form of GIBIM-P5S9K peptide within PLGA or polylactic acid exhibited around 20 times stronger antimicrobial efficacy against methicillin-resistant *S. aureus* (MRSA), *P*. aeruginosa, and *E. coli* when compared to the free peptide.

In addition, gold NPs have been increasingly applied in the field of HDPs (175). Comune et al. (171) demonstrated that LL-37 conjugated with gold NPs (via an additional cysteine residue at the C-terminus of LL-37) demonstrated superior *in vitro* and *in vivo* wound healing properties compared to LL-37 alone. This was attributed to the prolonged phosphorylation of epidermal growth factor receptor (EGFR) and extracellular-signal-regulated kinase (ERK)1/2, which increased the migration of keratinocytes. Gold NPs have also been used as nanocarriers for other HDPs such as Esc(1-21), a derivative of a frog skin HDP called esculentin-1a (176). Compared to Esc(1-21), it was found that the conjugated form of Esc(1-21) with gold NPs via a poly-ethylene glycol linker improved the antimicrobial efficacy against *P. aeruginosa* by around 15-fold, with increased resistance to proteolytic degradation (176). Certain peptides have also demonstrated self-assembling ability as a nanocarrier for drug delivery (166), though this is beyond the scope of our review.

### Smart Design Using Artificial Intelligence (AI) Technology

AI serves as one of the major breakthroughs in the mankind's history. Long been deployed in the automobile and technology industries, AI has only started gaining traction in the field of science and medicine, owing to the advancement in computer power, availability of big data, publicly available neural networks, and improvement in AI algorithms using machine learning and deep learning (177–180). In view of the infinite chemical space and complex SAR of natural and synthetic HDPs, AI serves as an attractive solution to identify and predict novel peptide sequences with potentially good antimicrobial efficacy (181–184).

To date, a number of machine learning algorithms such as artificial neural network (ANN) (34, 182), support vector machine (SVM)-based classifier (34, 183, 184), quantitative matrices (34), and fuzzy K-nearest neighbor (FKNN) (185) have been developed to search for the ideal synthetic HDPs. Cherkasov et al. (182) trained an atomic-based QSAR model using ANN and inductive chemical descriptors based on two large 9-mer peptide libraries. The model was then tested against 200 peptides that were chosen from a virtual library of 100,000 random 9-mer peptides. The model not only successfully predicted the antimicrobial efficacy of the synthetic peptides but also identified potent peptide candidates (HHC-10 and HHC-36) which were highly active against a range of Gram-positive and Gram-negative superbugs, with low risk of toxicity (182).

On the other hand, Lee et al. (183) developed a SVM-based classifier coupled with Pareto-optimization (186) to deduce the functional and structural similarities of alpha-helical HDPs. By employing antimicrobial assays and small-angle X-ray scattering, it was found that the SVM distance to hyperplane σ correlated strongly with the ability of HDP in generating a negative Gaussian curvature (NGC), which is commonly responsible for the membrane disruption mechanism of HDP (183). Subsequently, Yount et al. (184) were able to identify a unifying physicochemical characteristic of alpha-helical HDPs in a 3-dimensional space, termed the alpha-core signature, using knowledge-based annotation and pattern recognition analysis of bioinformatics databases. The antimicrobial efficacy of this alpha-core signature (i.e., the ability to induce NGC) was further validated with the previously developed SVM-based classifier (184).

## Discussion and Future Directions

Since the serendipitous discovery of HDPs in nature during early 1980s, immense research effort have been invested in realizing the therapeutic potentials of HDPs in clinic (7). In this review, we provided a comprehensive overview on eight key strategies (with examples) in improving and translating the therapeutic potentials of HDP-based treatment from bench to bedside. Moreover, we summarized HDP derivatives that are currently in the development pipeline.

### Lessons From Previous Experience

So far, a number of HDP-based treatment have entered advanced (phase II/III) clinical trials (Table 1) but none had reached the market due to regulatory hurdles (27, 187). Nevertheless, many lessons have been learnt from past experience. One of the notable examples (as described above) is MSI-78 or pexiganan, a magainin-derived HDP, which did not obtain the FDA approval after failing to demonstrate any superiority to the normal standard wound care with oral ofloxacin for infected diabetic foot ulcers in two phase 3 trials (188). Although the discouraging results have painted a gloomy outlook for HDP-based treatment at that time, a closer look at the development pathway of MSI-78 has shed light on the plausible reasons accounting for the failure. First, although the molecule demonstrated a broad-spectrum antimicrobial activity against 3,109 clinical isolates (with an average MIC_90_ of 32 μg/ml or less) (63), the activity remained considerably weaker than the conventional antibiotics (62). Second, peptide-based treatment including MSI-78 are more susceptible to proteolytic degradation when compared to the conventional small molecule antibiotics. That means HDP-based treatment need to be administered in a higher concentration to achieve the intended *in vivo* efficacy, which could inevitably lead to increased host toxicity. In addition, MSI-78 exhibits several favorable properties over conventional antibiotics, including low risk of developing AMR and good activity against MDR isolates (63), but the phase 3 clinical trials of MSI-78 were conducted for mild infective diabetic foot ulcers which did not fully capitalize on these strengths. This highlights the importance of setting the right research question during the development of HDP-based treatment.

Learning from the previous (unsuccessful) experience, a plethora of strategies have been proposed and attempted to overcome the inherent limitations of HDP-based treatment, with enhanced antimicrobial efficacy, proteolytic stability, and cell selectivity (for microbial cells). Although the design of ideal HDPs is not governed by a single overarching rule (7), it is apparent from the literature that peptide design guided by the fundamental principles and systematic SAR analysis is able to yield potential efficacious peptide candidates with desired properties (189). In fact, *de novo* designed synthetic peptides were successfully developed purely based on Arg and Trp with 50% hydrophobicity and demonstrated significantly antimicrobial and anti-biofilm efficacies against MDR staphylococci (190).

### Proposed Strategy in Designing and Developing HDP-Based Treatment

**Figure 1:**
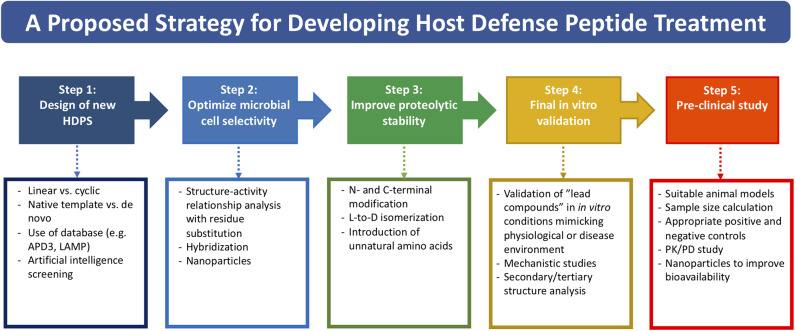
A potential strategy for streamlining the drug discovery and developmental pathway of HDP-based treatment, covering from designing of new HDP treatment to conducting well-designed pre-clinical studies.

Based on the literature and our experience, we propose a potential strategy in streamlining the drug discovery and development pathway of HDP-based treatment, starting from designing new HDP treatment to conducting well-designed pre-clinical studies (Figure 1). So far, more than 3,000 naturally occurring and synthetic HDPs (with reported antimicrobial and/or non-antimicrobial functions) have been discovered (8, 9); therefore, it would be a good strategy to use an existing template with proven effect as a starting point for designing a new HDP-based treatment. Alternatively, employing artificial intelligence technology in predicting potentially efficacious molecules could be utilized. Once a starting template is identified (either a linear peptide or a cyclic peptide), systematic SAR analysis of the sequence via rational substitution of specific residues is required to optimize the antimicrobial efficacy and cell selectivity toward microbial cells. If hybridization strategy is used, functioning sequence of each single peptide should be first determined before being hybridized. This is then followed by further SAR analysis to determine the optimal sequence.

Once the efficacy and safety are optimized, the next hurdle is to overcome the issue of proteolytic degradation, which could be achieved through the strategies (either singularly or in combination) mentioned in Table 2 and Figure 1. However, it is noteworthy to mention that the beneficial effects of these modifications may be unique to specific HDPs. In addition, antimicrobial efficacy and/or microbial cell selectivity of the HDPs may also be affected during the modification. Subsequently, the potential lead compound should be validated in *in vitro* conditions mimicking the physiological or host disease environment. For instance, when designing HDP-based treatment for corneal infection, the designed HDPs should be tested in tear fluid or in salt of physiological concentration, which are known to affect the efficacy and stability of HDPs. The findings enable a better prediction of the *in vivo* results and help minimize the unnecessary use of animals (191). Finally, well-designed pre-clinical studies need to be performed with appropriate sample size calculation and positive/negative controls, which will increase the success rate of clinical trials. For example, efficacy of the designed HDP needs to be compared with antibiotic treatment that reflects the current clinical practice for the disease of interest. Otherwise, subsequent clinical trials are likely to fail and necessary regulatory approval will not be obtained.

**Table 2:** Summary of different strategies in translating the therapeutic potentials of host defense peptides (HDPs).

**Methods**	**HDPs template**	**Strategies**	**Biological effects**
**Residue substitution**			
Lee et al. (40)	HP ribosomal protein 1	Pro substitution	Increased antimicrobial efficacy
Wang et al. (86)	LL-37	Ala/Val substitution	Increased antimicrobial efficacy
Blondelle et al. (37)	Melittin	Trp substitution	Reduced host tissue toxicity
**Hybridization**			
Wei et al. (117)	Cecropin and LL-37	Hybridization	Increased antimicrobial efficacy and reduced host tissue toxicity
Wu et al. (118)	Melittin and LL-37	Hybridization	Increased antimicrobial efficacy and reduced host tissue toxicity
Boman et al. (112)	Cecropin and melittin	Hybridization	Improved antimicrobial efficacy and reduced host tissue toxicity
**Unnatural AA**			
Arias et al. (101)	Indolicidin	Ornithine, DAB, DAP, Agb, and hArg	Improved antimicrobial activity against GN and proteolytic stability
Clemens et al. (100)	Cecropin and magainin	Ornithine	Good antimicrobial and anti-biofilm efficacies against GP and GN
Hicks et al. (103)	Magainin	Tic-Oic	Increased antimicrobial activity against GP, GN and mycobacterium and reduced host tissue toxicity
**L-to-D isomerization**			
Jia et al. (131)	Polybia-CP	LDI	Improved proteolytic stability and reduced host tissue toxicity
Manabe et al. (132)	Sapesin B	LDI	Improved antimicrobial efficacy against GP, GN and fungi
Carmona et al. (130)	Pandinin 2	LDI	Reduced host tissue toxicity
**C- and N- terminal modifications**			
Saikia et al. (139)	MreB	N-acetylation	Improved antimicrobial efficacy in salt
Falciani et al. (148)	M33	C-pegylation	Increased proteolytic stability
Dennison and Phoenix (143)	Modelin-5	C-amidation	Improved stabilization of alpha-helix and antimicrobial efficacy
**Cyclization**			
Mwangi et al. (161)	Cathelicidin-BF	Cyclization	Increased antimicrobial and antibiofilm efficacies against MDR-GN and good proteolytic stability
Scudiero et al. (160)	HBD-1 and−3	Cyclization	Increased proteolytic stability
Fernandez-Lopez et al. (154)	*De novo*	Cyclization of D,L-alpha peptides	Increased antimicrobial efficacy
**Incorporation with nanoparticles**			
Comune et al. (171)	LL-37	Gold NP	Improved wound healing
Casciaro et al. (176)	Esculentin-1a	Gold NP	Improved antimicrobial efficacy, wound healing, and proteolytic stability
Chereddy et al. (169)	LL-37	PLGA NP	Improved wound healing
**Smart design with artificial intelligence technology**			
Yount et al. (184)	5,200 12-mer peptide sequence	SVM-based classifier	Identification of a unifying alpha-core signature of peptide with good correlation with ability to generate NGC
Lee et al. (183)	572 alpha-helical peptides	SVM-based classifier	Accurate prediction of peptide ability to generate NGC
Cherkasov et al. (182)	Random 9-mer peptide database	QSAR model using ANN	Generation of highly active synthetic peptides against MDR GP and GN, with low toxicity

*Three representative examples are provided for each strategy, in order of chronology*.

*HP, Helicobacter pylori; GP, Gram-positive bacteria; GN, Gram-negative bacteria; DAB, 2,4-diamino-butyric acid; DAP, 2,3-diamino-propionic acid (DAP); Agb, (S)-2-amino-4-guanidinobutyric acid; hArg, homo-arginine; Tic-Oic, tetrahydroisoquionolinecarboxylic acid-octahydroindolecarboxylic acid dipeptide; HBD, Human-beta-defensin; PLGA, Poly lactic-co-glycolic acid; SVM, support vector machine; NGC, Negative Gaussian curvature; ANN, Artificial neural network; MDR, Multidrug resistant*.

Ideally, it is best to optimize the previous steps before progressing to the next step. For instance, validating the potential lead compound in *in vitro* conditions mimicking physiological environment is crucial before proceeding to pre-clinical studies. Modification strategies proposed in each step may also be applicable to other steps. For example, introduction of unnatural AAs primarily improves the proteolytic stability but may also enhance the antimicrobial efficacy (101), and incorporation of HDPs with nanoparticles may reduce host toxicity as well as improve bioavailability. In addition, several strategies may be employed in combination to achieve the intended therapeutic effect and stability.

There are also increasing reports examining and exploiting the strategy of using peptide-antibiotic combination to counter AMR, increase the lifespan of conventional antibiotics and HDPs, as well as to reduce the undesired toxicity to host tissues (88, 192–194). The synergistic effect of peptide-antibiotic combination treatment is likely attributed to the different underlying mechanism of action whereby the membrane perturbation effect of peptides facilitates the passive diffusion of conventional antibiotics into the cells for intracellular targeting action (192).

### Risk of AMR Related to HDP

Although HDP-based treatment has long been envisioned as a novel solution to tackle AMR, emerging evidence are suggesting that HDPs could also develop AMR, albeit with a lower risk than the conventional antibiotics (7, 195). Spohn et al. (196) have highlighted the influence of physicochemical characteristics of HDPs, including the proportion of polar AAs, cationicity, and hydrophobicity, on the risk of developing HDP-related AMR, thereby providing invaluable insights into the design of future HDPs. Reassuringly, cross resistance between HDPs was found to be limited to those with similar modes of action, underscoring the importance and necessity of having HDPs with different antimicrobial mechanisms within the therapeutic armamentarium of antimicrobials (197).

With the advancement in peptide design strategy, synthesis techniques and AI technology, it is hopeful that clinical deployment of HDP-based treatment for a range of diseases will soon become a reality. However, further studies will need to be conducted to decipher the mechanism of HDP-related AMR in order to prepare for the potentially self-perpetuating vicious cycle of AMR in the future.

## Method of Literature Search

Electronic databases, including MEDLINE (January 1950–March 2020) and EMBASE (January 1980–March 2020), were searched for relevant articles related to host defense peptides. Keywords such as “host defense peptide,” “antimicrobial peptide,” “hybrid,” “cyclization,” “unnatural amino acid,” “D-amino acid,” “nanotechnology,” “nanoparticles,” “artificial intelligence,” and “machine learning” were used. Only articles published in English were included. Bibliographies of included articles were manually screened to identify further relevant studies. The final search was updated on 31 March 2020.

## Author Contributions

DT and IM: conception and design of work, data collection and interpretation, drafting the manuscript, and final approval of the work. RB and HD: data interpretation, critical revision, and final approval of the work. RL: conception and design of work, data interpretation, critical revision, and final approval of the work.

## Conflict of Interest

HD receives travel honorarium from Thea, Dompe, and Santen and has shares in GlaxoSmithKline and NuVision Biotherapies. The remaining authors declare that the research was conducted in the absence of any commercial or financial relationships that could be construed as a potential conflict of interest.
